# Supplementary material for: Clinical impact of respiratory virus in pulmonary exacerbations of children with Cystic Fibrosis
Source: PLoS One. 2020 Oct 28;15(10):e0240452. doi: 10.1371/journal.pone.0240452 (PMC7592759; doi:10.1371/journal.pone.0240452)
Supplement: Attachment — Submitted filename: Response do Reviewers.docx [file pone.0240452.s002.docx]

**Response to Reviewers**

Dear Dr. Omri,

Thank you for your considerations and for the opportunity to submit a revised version of the manuscript *“Clinical impact of respiratory virus in pulmonary exacerbations of children with Cystic Fibrosis”* to PLOS ONE. We appreciate the insightful comments provided by you and the reviewers, which we believe have brought valuable improvements to our paper. We have incorporated most of these suggestions, and respond below with the changes made.

1. **Editor’s Comments**

This is a very simple retrospective analysis of data that I think is flawed in combining static, patient-specific features with variable event-specific features. I would encourage evaluation by a statistician.

We thank you for your considerations. The study was reviewed by a statistician and patient-specific features, such as gender and genetics were separately described in a different table (Table 2). Although this observational analysis may seem simple, few articles use estabileshed comparable tools in defining pulmonary exacerbation events and grading their severity, and we believe the study has a legitimate contribution to add. Furthermore, since CF is a rare disease, very few published studies on pulmonary exacerbations can recruit a large number of patients.

Please ensure that your manuscript meets PLOS ONE's style requirements, including those for file naming.

File naming was corrected as per PLOS ONE’s style requirements.

You indicated that you had ethical approval for your study. In your Methods section, please ensure you have also stated whether you obtained consent from parents or guardians of the minors included in the study or whether the research ethics committee or IRB specifically waived the need for their consent.

Consent information was obtained from all parents/guardians, and this information was included in the Methods Section.

We note that you have indicated that data from this study are available upon request. PLOS only allows data to be available upon request if there are legal or ethical restrictions on sharing data publicly.

We have included a minimal anonymized data set from the Study as a Supporting Information File.

2. **Reviewer #1`s Comments**

Although the manuscript is an original work, it is more like an observational study, in which the findings reported are very similar to previously published articles, at least in some form. The conclusion of this study is very similar to other published reports and thus it does not fulfill the criteria of Replication studies described in the Plos One Guidelines for Reviewers. I suggest to submit it as an 'Observational study' instead of 'original article'.

We thank you for your considerations, but we checked with the journal office and PLOS ONE does not have a 'Observational study'  article type. Therefore we resubmitted the manuscript as a "Research Article", which include observational studies.

3. **Reviewer #2`s comments**

The authors have completed a prospective collection of samples and data on a cohort of subjects with cystic fibrosis. They have determined the viral infection at the time of pulmonary exacerbation and identified factors that differentiate those that are virus positive compared with virus negative. The authors have provided a comprehensive introduction and discussion with reference to the established literature. They authors have used well established assessment tools in CF research. As such, the study provides some useful information. I have a few major and minor comments to make that could be addressed by the authors.
Major
Since the number of positive subjects is quite limited, I would like to know the power to detect statistical differences between virus positive and negative groups for each outcome. This could be added as an online supplement. I am concerned this study does not have the statistical power to detect differences based on the subject numbers and that is why so few outcomes are significant.
We appreciate your considerations. The study was reviewed by a statistician and we have included a minimal anonymized data set from the Study as a Supporting Information File.

If a result is not statistically significant (or even close to p-0.05) then there is no relationship. For example, the authors claim that they virus positive group were younger, but statistically they were not (p=0.3), therefore they are NOT younger. This is described in the results and the discussion. This also includes fever, fatigue antibiotic prescription, P. aeruginosa prevalence, acquisition of P. aeuginosa, intermittent or chronic P. aeruginosa in the whole group or the children <5 years of age (outlined in the discussion).

Thank you for your correction. The study was reviewed by a statistician and the discussion was rewritten. All sections describing relationships that were not statistically significant were reviewed and corrected to show that in fact no relationship could be found. The limited size of the sample was also stressed as a potential limitation for statistical analysis.

It would be helpful if the authors identified which previous studies mentioned in the discussion (for example: when the authors are discussing the lack of difference in CF severity between virus positive and negative groups) were of a similar age and which were from older children.

We appreciate you pointing this out. In fact, these previous studies included adolescents and adults, and we have now pointed this out in the discussion (“However, median age in these studies was older, including adolescents and adults”).

Minor
Although the article is quite well written and perfectly understandable – the article requires further editing for English.

The manuscript was thoroughly reviewed and edited for English.

It would be helpful if standard acronyms were used such as URTI (upper respiratory tract illness) instead of URTS.

Since the Fuchs criteria for PE uses the term “change in sinus discharge” we substituted URTS for this term, in order to keep uniformity throughout the manuscript.

4. **Reviewer #3’s Comments**

The manuscript by Correa Meyer et al describes an analysis of respiratory virus infection in persons with CF during episodes of pulmonary exacerbation (PE). The aim of the study was to compare virus-positive and virus-negative groups in regards to clinical manifestations, severity of PE and bacterial infection. The authors conclude that prospective studies are needed to assess the impact of viral infection in CF. A concern with the design and analysis of data in this study is that patient-specific (static) and exacerbation event-specific (variable) characteristics are combined and compared (Table 2). That is, some patient-specific features (eg CFTR genotype, gender pancreatic insufficiency) are counted as independent features for each episode of exacerbation. This seems to introduce confounding. Eg, if an individual heterozygous for F508del had three exacerbation episodes, but by virtue of lack of siblings or age, none of these were associated with viral infection, the three episodes would inflate the number of virus-negative events in the F508del heterozygous group… supporting that viral infection is less common in persons heterozygous for F508del. A more robust multivariable analysis with attention to interactions between variables seems to be required.

We appreciate you pointing this out. In fact, we reviewed the study with a statistician and excluded patient-specific features from the comparison of virus positive and virus negative exacerbations. These features were separately described in Table 2. Due to the limited size of the sample a more robust multivariable analyses was not possible.

On a more minor note: There are implications of causality, when associations are all that can be assessed. Eg: Abstract: do exacerbations cause morbidity in CF … or are these events a sign of morbidity? Eg: Intro: do exacerbations impact disease progression…or are these events a manifestation of lung disease progression? Eg, have the studies cited (refs 3-15) show that viral infection is ‘responsible for’ exacerbation events?

We agree that in the first two scenarios (exacerbations vs. morbidity and exacerbations vs. disease progression), the exacerbations may be found to be cause or consequence, and therefore have rewritten these sections. As for respiratory virus being responsible for exacerbation events, we have re-written the sentence in order to differentiate studies that only showed the prevalence of virus in exacerbations from the studies that showed a significant association of virus with PE events (“ several studies have demonstrated a large prevalence of virus in PE events^3-15^, showing significant association between virus detection and PE symptoms^5,8,9,12,15^.”).

5. **Reviewer #4s Comments**

This is a study from Brazil in pediatric patients with CF that explores whether viral DNA/RNA can be found during a CF exacerbation. The authors found that 36% of 70 respiratory CF exacerbations were associated with retrieval of viral genetic material from NP swabs. Comments:
Unfortunately this is a relatively small study, and statistical power is quite limited. Therefore the investigators were not able to show significant differences between viral-associated exacerbations and non-viral exacerbations. I think that the study abstract and conclusions need to stress lack of statistical power as a major limitation of this study, this may explain why significant differences between viral-associated exacerbations and non-viral exacerbations was not found.
We agree this is a potential limitation, and have re-written these sections in order to stress this.

The fact that viral exacerbations were associated with upper resp tract symptoms is of course not surprising, and this may need to be made clearer in the abstract and conclusions.

We agree that this is not surprising and have re-written this information (“as would be expected has been described by other authors^8,9^”).

Tables 2 and 5 are far too long and hard to read and all statistical comparisons are non-significant. Please limit these tables to important variables only- please shorten them to 5-6 variables.

The tables were shortened to include only more relevant variables.

The discussion should include mention of a study that is highly relevant : Chin M, De Zoysa M, Slinger R, Aaron SD. Acute effects of viral respiratory tract infections on sputum bacterial density during CF pulmonary exacerbations. Journal of Cystic Fibrosis, 14: 482-489, 2015.

Thank you for the suggestion, the mentioned article was included in the discussion (“On the other hand, a study by Chin et al didn’t find an increase in *Pseudomonas* density in sputum during viral infections^30^. As in most previous studies^9,10,22,31^ we found no difference of pseudomonas prevalence in patients with viral detection”).

We hope the revised manuscript will better suit PLOS ONE but are happy to consider further revisions, and we thank you for your continued interest in our research.

On behalf of the co-authors,

Viviane Mauro Corrêa Meyer
